# First-in-human, open-label dose-escalation and dose-expansion study of the safety, pharmacokinetics, and antitumor effects of an oral ALK inhibitor ASP3026 in patients with advanced solid tumors

**DOI:** 10.1186/s13045-016-0254-5

**Published:** 2016-03-10

**Authors:** Tianhong Li, Patricia LoRusso, Michael L. Maitland, Sai-Hong Ignatius Ou, Erkut Bahceci, Howard A. Ball, Jung Wook Park, Geoffrey Yuen, Anthony Tolcher

**Affiliations:** Division of Hematology/Oncology, University of California Davis Comprehensive Cancer Center, 4501 X St #3016, Sacramento, CA 95817 USA; Karmanos Cancer Institute, Wayne State University, Detroit, MI USA; Section of Hematology/Oncology, Committee on Clinical Pharmacology and Pharmacogenomics, University of Chicago Medicine, Chicago, IL USA; Chao Family Comprehensive Cancer Center, University of California Irvine School of Medicine, Orange, CA USA; Astellas Pharma Global Development, Northbrook, IL USA; South Texas Accelerated Research Therapies (START) Center for Cancer Care, San Antonio, TX USA; Present address: Yale Smilow Cancer Center, New Haven, CT USA

**Keywords:** ASP3026, Neoplasms, ALK inhibitor, Phase I, Pharmacokinetics

## Abstract

**Background:**

ASP3026 is a second-generation anaplastic lymphoma kinase (ALK) inhibitor that has potent in vitro activity against crizotinib-resistant *ALK*-positive tumors. This open-label, multicenter, *first-in-human* phase I study (NCT01284192) assessed the safety, pharmacokinetic profile, and antitumor activity of ASP3026.

**Methods:**

Advanced solid tumor patients received oral ASP3026 in 3 + 3 dose-escalation cohorts at doses of 25–800 mg once daily in 28-day cycles. The endpoints were to identify the maximum tolerated dose (MTD), the recommended phase II dose (RP2D), and the pharmacokinetic profile of ASP3026. A phase Ib expansion cohort enrolled patients with metastatic, crizotinib-resistant *ALK*-positive solid tumors at the RP2D, and response was evaluated by RECIST 1.1.

**Results:**

The dose-escalation cohort enrolled 33 patients, including three crizotinib-resistant, *ALK*-positive patients, and the dose-expansion cohort enrolled another 13 crizotinib-resistant, *ALK*-positive non-small cell lung cancer (NSCLC) patients. ASP3026 demonstrated both linear pharmacokinetics and dose-proportional exposure for area under the plasma concentration–time curve and maximum concentration observed with a median terminal half-life of 35 h, supporting the daily dosing. Grade 3 rash and elevated transaminase concentrations were dose-limiting toxicities observed at 800 mg; hence, 525 mg daily was the MTD and RP2D. The most common treatment-related adverse events were nausea (38 %), fatigue (35 %), and vomiting (35 %). Among the 16 patients with crizotinib-resistant *ALK*-positive tumors (15 NSCLC, 1 neuroblastoma), eight patients achieved partial response (overall response rate 50 %; 95 % confidence interval 25–75 %) and seven patients (44 %) achieved stable disease.

**Conclusions:**

ASP3026 was well tolerated and had therapeutic activity in patients with crizotinib-resistant *ALK*-positive advanced tumors.

**Trial registration:**

ClinTrials.gov: NCT01284192

## Background

In recent years, aberrant expression of anaplastic lymphoma kinase (ALK) tyrosine kinase receptor has emerged as a relevant biomarker and therapeutic target for a number of solid tumors [1, 2]. Different types of alterations in the *ALK* gene have been implicated in human cancer tumorigenesis, and different tumor types have different structural alterations in the *ALK* gene [3, 4]. In non-small cell lung cancer (NSCLC), echinoderm microtubule-associated protein-like 4 (*EML-4*) and kinesin family member 5B (*KIF5B*) account for the majority of *ALK* gene rearrangement lung cancer [5]. The presence of ALK gene rearrangement defines ~3–13 % of NSCLC [6–10] that are highly sensitive to the first-generation ALK tyrosine kinase inhibitor (TKI), crizotinib. Crizotinib (250 mg twice daily) has high efficacy in patients with NSCLC harboring this oncogenic kinase, with overall response rates of >50 % [11–13]. Crizotinib has been established as the standard first-line treatment for patients with advanced ALK-positive NSCLC [13]. The current National Comprehensive Cancer Network (NCCN) guideline recommend crizotinib use in patients with advanced NSCLC harboring *ALK* gene rearrangement [14]. However, almost all patients develop resistance, typically within 10 months [11, 13, 15, 16].

The most common molecular mechanisms of resistance include amplification of the *ALK* fusion gene, development of resistance mutations, and activation of alternative or bypass signaling pathways or progression in the CNS [17]. One strategy to overcome crizotinib resistance is to develop potent small molecule TKIs for *ALK*-rearranged genes and/or specifically target the common resistant mutations, such as gatekeeper mutation L1196M [17].

ASP3026 is a selective, ATP-competitive, second-generation ALK TKI that was identified through a medicinal chemistry campaign designed to obtain compounds with a better pharmacologic profile compared with crizotinib. The kinase selectivity of ASP3026 was evaluated and compared with that of crizotinib against a panel of 86 tyrosine kinases [18]. ASP3026 at 1 μmol/L inhibited 11 tyrosine kinases by more than 50 %, with the highest selectivity for ALK, ROS1, and ACK kinases, showing that the kinase selectivity of ASP3026 differed from crizotinib. ASP3026 was more selective for FRK, YES, ACK, TNK1, and EGFR (L858R), whereas crizotinib had higher selectivity for MET, RON, LCK, JAK2, MUSK, TRKs, TYRO3, AXL, MER, and EPHs [18]. ASP3026 fits within the ATP-binding pocket of both wild-type and L1196M ALK kinase domains and inhibits their kinase activities with IC_50_ values of 10 and 32 nmol/L, respectively. By contrast, crizotinib fits within the ATP-binding pocket of wild-type ALK kinase domain but not the L1196M ALK kinase domain. Thus, crizotinib displays tenfold weaker activity for the mutated *EML4-ALK* compared with the wild-type *ALK* gene [18]. In mice bearing subcutaneous and intracranial xenograft tumors, ASP3026 has potent antitumor activity against both wild-type *ALK* and *EML4-ALK* L1196M xenograft tumors compared with crizotinib [18]. ASP3026 also has a higher tissue-to-plasma ratio compared with crizotinib, which could translate into a wide therapeutic margin between efficacious and toxic doses [18]. Preclinical data indicated that ASP3026 may have potential therapeutic effects for patients with crizotinib-resistant *ALK*-positive NSCLC and potentially for patients with other cancer types of *ALK*-driven tumors.

We conducted this phase I dose-escalation trial to evaluate the safety and pharmacokinetics (PKs) of ASP3026 as an oral single agent in patients with advanced solid malignancies. A planned phase Ib dose-expansion cohort at the recommended phase II dose (RP2D) was conducted to evaluate the tumor response of ASP3026 in patients with metastatic *ALK*-positive NSCLC who progressed on crizotinib.

## Methods

### Clinical study summary

The clinical trial design was a phase I, multicenter, open-label, dose-escalation and dose-expansion study (NCT01284192) of ASP3026 in patients with advanced malignancies. The study was conducted in accordance with all applicable regulatory requirements and had institutional review board approval prior to study initiation at participating institutions. Written informed consent was obtained from all patients prior to the initiation of any study-specific procedures.

### Patient population

Adult patients (≥18 years of age) with histologically or cytologically confirmed diagnosis of relapsed/refractory tumor were included in the dose-escalation study. Patients had to have Eastern Cooperative Oncology Group (ECOG) performance status ≤2, adequate life expectancy >12 weeks, be non-child bearing (or be using protocol-specified contraceptive measures), and be able to swallow oral medications. Additional criteria for patients with *ALK* abnormalities in the dose-escalation phase included patients to be positive for *ALK* abnormalities (by any molecular method including, but not limited to, polymerase chain reaction, direct sequencing, in situ hybridization, or be previously confirmed by fluorescence in situ hybridization), to not have symptomatic brain metastases, to not to be taking >5 mg prednisone daily, or to not require hepatic enzyme-inducing anti-seizure medication.

Inclusion into the dose-expansion cohort required patients to have *ALK*-positive tumors that had progressed on crizotinib. Key exclusion criteria for the dose-escalation cohort included patients with leptomeningeal involvement (as assessed through medical history review or through physical examination), inadequate bone marrow, renal and/or hepatic function, and a known history of long QT syndrome. Brain magnetic resonance imaging (MRI) was carried out for all dose-expansion patients and for all dose-escalation patients known to have brain metastases at screening. For patients with baseline brain metastases, MRI was performed at the end of cycle 2 and then every 2 cycles thereafter. Only *ALK*-positive subjects were eligible for the dose-expansion cohort; *ROS1* was not included.

### Study design and treatments

The study was divided into two parts: dose escalation and dose expansion. Dose escalation used a traditional 3 + 3 dose-escalation design. Cycles of treatment were every 28 days with continuous dosing of ASP3026. Patients were followed-up for safety assessments 30 days (±7 days) after the last ASP3026 dose. The starting dose for ASP3026 was 25 mg administered orally once daily. Dose escalation proceeded to the next seven cohorts of 50, 75, 125, 200, 325, 525, and 800 mg. The first patient in each dose-escalation cohort was evaluated for dose-limiting toxicities (DLTs) in cycle 1, day 4. If no DLTs were reported by the investigator, the remaining patients in the cohort were enrolled. Therapeutic concentrations were projected to be reached above 325 mg. The protocol allowed that and subsequent doses to be expanded to enroll an additional three patients who were known to have tumors tested positive for *ALK* abnormalities once safety was established in the first three subjects for that cohort. To further address the antitumor effects and the safety of ASP3026 in patients who progressed on crizotinib, the dose-expansion part of the study focused on crizotinib-refractory *ALK*-positive NSCLC patients.

The primary objectives of the study were to determine the safety and tolerability of ASP3026 in patients with advanced malignancies and to determine the maximum tolerated dose (MTD) and RP2D for ASP3026. The MTD was defined as the highest dose of ASP3026 at which <33 % of patients experienced a DLT during cycle 1. Secondary objectives were to determine the PKs and antitumor activity of ASP3026.

### Safety/tolerability assessments

The safety and tolerability of ASP3026 were assessed by adverse events (AEs) (graded based on NCI-CTCAE v4.03), laboratory tests, vital signs, electrocardiograms, and clinical observations.

Dose-limiting toxicity criteria were grade 4 neutropenia lasting ≥7 days, febrile neutropenia, grade 3 thrombocytopenia, grade 3 non-hematologic toxicity, except for nausea/vomiting or diarrhea (nausea/vomiting or diarrhea was considered a DLT in patients who had grade 3 toxicity for ≥3 days or grade 4 toxicity of any duration), and any study drug-related toxicity resulting in treatment delay >2 weeks or discontinuation of treatment at the assigned dose level.

### Pharmacokinetic assessments

Plasma PKs samples were taken on days 1, 2, 8, 15, 22, and 28 (±2 days) of cycle 1. On day 1, the following PK parameters were assessed for ASP3026: area under the plasma concentration–time curve (AUC_24_), AUC_last_, maximum concentration observed (*C*_max_), and time of maximum concentration observed (*t*_max_). On day 28, AUC_tau_, *C*_max_, *C*_trough_, *t*_max_, CL/F, V_z_/F, and *t*_½_, calculated based on the ratio of accumulation for AUC (day 28 AUC_tau_/day 1 AUC_24_) (Rac [AUC]), were assessed. Cumulative effects on steady-state ASP3026 plasma levels were assessed by comparing *C*_trough_ on day 28 divided by *C*_trough_ on days 8, 15, and 22.

### Antitumor assessments

Solid tumor assessment was based on Response Evaluation Criteria in Solid Tumors (RECIST) v1.1. For target lesions, complete response (CR) was defined as disappearance of all target lesions and pathologic lymph nodes with a reduction in short axis to <10 mm. Partial response (PR) was defined as a ≥30 % decrease in the sum of diameters of the target lesions, taking as reference the baseline sum of diameters. Progressive disease (PD) was defined as a ≥20 % increase in the sum of diameters from the smallest sum on the study and the sum of diameters to be ≥5 mm from the smallest sum on study. Stable disease (SD) was defined as neither PR nor PD.

For patients in the dose-expansion cohort with evaluable tumor diameter, the best tumor reduction was calculated as the decrease from baseline in the sum of the target lesions.

### Statistical analyses

For continuous variables, descriptive statistics included the number of patients, mean (standard deviation), median, and range. For antitumor assessments, the number and percentage (95 % CI) of patients with CR, PR, SD, and PD were summarized. The overall best tumor response was also summarized. For the objective response rates, the exact confidence interval of response was calculated only for the dose-expansion cohort using the Clopper–Pearson method. For continuous PK parameters, the coefficient of variation was calculated. For *C*_max_ and AUC, geometric mean was calculated. All data processing and analyses were performed using SAS® Version 9.1.3 or higher. The safety analysis set was defined as patients who received at least one ASP3026 dose. The PK analysis set was defined as patients who received at least one dose of ASP3026 and provided the values of drug concentrations for at least one time point.

## Results

### Baseline patient characteristics

Enrollment began on 11 January 2011 and closed on 28 June 2013. The data cut-off date was 19 February 2014. Forty-six patients (33 patients in the dose-escalation cohort; 13 in the dose-expansion cohort) were included in the current analyses.

Table 1 summarizes patients’ demographics. For all study patients, 22 (48 %) were men and the median (range) age was 61 (19–77) years. In the dose-escalation cohort, the most common primary tumor types were breast and lung adenocarcinoma (both *n* = 4), leiomyosarcoma and adenocarcinoma (unspecified primary) (both *n* = 3), and bile duct, colon, and ovarian cancer (each *n* = 2). The median (range) duration of prior chemotherapy/targeted therapy was 46 (1–171) days. Patients were not mandatorily screened for brain metastases at baseline in the dose-escalation cohort.

**Table 1:** Patient demographics and baseline disease characteristics

Parameter/statistics	Dose-escalation cohort	Dose-expansion cohort (*ALK*-positive)	Both cohorts
	Total (*n* = 30)^a^	525 mg (*n* = 16)^b^	All patients (*n* = 46)
Sex, *n* (%)			
Male	14 (47)	8 (50)	22 (48)
Female	16 (53)	8 (50)	24 (52)
Race, *n* (%)			
White	25 (83)	1 (6)	26 (57)
Black or African American	5 (17)	14 (88)	19 (41)
Asian	0	1 (6)	1 (2)
Age (years)			
Mean (standard deviation)	61.6 (9.6)	51.1 (11.8)	57.9 (11.5)
Median (range)	64 (44–77)	51 (19–71)	61 (19–77)
Weight (kg), mean (standard deviation)	80.3 (20.4)	75.0 (11.6)	78.5 (17.9)
ECOG performance status, *n* (%)			
Grade 0	6 (20)	9 (56)	15 (33)
Grade 1	19 (63)	7 (44)	26 (57)
Grade 2	5 (17)	0	5 (11)
Primary tumor type, *n* (%)			
Lung adenocarcinoma	4 (13)	7 (44)	11 (24)
NSCLC	0	6 (38)	6 (13)
Malignant lung neoplasm	0	2 (13)	2 (4)
Breast	4 (13)	0	4 (9)
Adenocarcinoma (unspecified primary)	3 (10)	0	3 (7)
Leiomyosarcoma	3 (10)	0	3 (7)
Colon	2 (7)	0	2 (4)
Bile duct	2 (7)	0	2 (4)
Ovarian	2 (7)	0	2 (4)
Other	10 (33)	1 (6)	11 (24)
Brain metastases history, *n* (%)	–	9 (56)	–
Prior radiation therapy, *n* (%)	18 (60)	14 (88)	32 (70)

*ALK* anaplastic lymphoma kinase, *ECOG* Eastern Cooperative Oncology Group, *NSCLC* non-small cell lung carcinoma, *UNK* unknown

^a^Excludes 3 *ALK*-positive patients

^b^Includes 3 *ALK*-positive patients from the dose-escalation cohort

In the 16 *ALK*-positive patients (including three *ALK*-positive patients from the dose-escalation cohort and 13 from the dose-expansion cohort), 15 NSCLC patients had *ALK* rearrangement and one neuroblastoma patient had an oncogenic *ALK* gene mutation F1174L [19–21]. Eight patients (50 %) were male, and the median (range) age of the patients was 51 (19–71) years (Table 1); nine patients (56 %) had brain metastases. All *ALK*-positive patients had progressed on crizotinib.

### Discontinuations, dose-escalation, and dose-limiting toxicities

All 30 enrolled patients (excluding three *ALK*-positive patients) discontinued treatment in the dose-escalation cohort (28 due to PD and two for other reasons [see “Safety/tolerability assessments” section]). At the time of data cut-off, 12 (75 %) discontinued treatment, 11 due to PD and one patient died in the dose-expansion cohort.

In the dose-escalation cohort, two patients receiving 800 mg ASP3026 experienced protocol-defined DLTs, probably related to the study drug, of increased aspartate aminotransferase and drug eruption (maculopapular rash on trunk, lower extremities, face, arm, and back), both grade 3. MTD was determined to be 525 mg, which was subsequently administered in the dose-expansion cohort.

### Adverse events

Within the dose-escalation and dose-expansion cohorts, 29 patients (97 %) and 16 patients (100 %) experienced ≥1 AE, respectively. Drug-related AEs were reported by 20 patients (67 %) in the dose-escalation cohort and 15 patients (94 %) in the dose-expansion cohort (Table 2).

**Table 2:** Summary of AEs possibly or probably related to study drug occurring in ≥2 patients in either cohort

	Dose-escalation cohort	Dose-expansion cohort (*ALK*-positive)	Both cohorts
	Total (*n* = 30)^a^	525 mg (*n* = 16)^b^	Total (*n* = 46)
Overall	20 (67)	15 (94)	35 (76)
Nausea	7 (23)	10 (63)	17 (37)
Vomiting	6 (20)	10 (63)	16 (35)
Fatigue	13 (43)	3 (19)	16 (35)
Decreased appetite	1 (3)	4 (25)	5 (11)
Diarrhea	3 (10)	2 (13)	5 (11)
Rash	0	3 (19)	3 (7)
Headache	1 (33)	2 (13)	3 (7)
Constipation	2 (7)	1 (6)	3 (7)
Peripheral neuropathy	0	2 (13)	2 (4)
Cataract nuclear	0	2 (13)	2 (4)
Periorbital edema	0	2 (13)	2 (4)
Blurred vision	0	2 (13)	2 (4)
Anemia	2 (7)	0	2 (4)
Increased blood creatinine	2 (7)	0	2 (4)

*ALK* anaplastic lymphoma kinase

^a^Excludes 3 *ALK*-positive patients

^b^Includes 3 *ALK*-positive patients from the dose-escalation cohort

Serious AEs (SAE) were reported by seven patients (23 %) in the dose-escalation cohort; one SAE was possibly related to the study drug (international normalized ratio [INR] increased that resolved after drug withdrawal). Serious AEs were reported by five patients (31 %) in the dose-expansion cohort; one SAE was possibly related to the study drug (abnormal liver function test that did not resolve after drug withdrawal) and another probably related to the study drug (keratoacanthoma that resolved after drug withdrawal). There were three deaths on study, all of which were due to PD and not related to the study drug.

Adverse events leading to drug discontinuation occurred in two patients in the dose-escalation cohort (one patient experienced a pulmonary embolism [not study drug-related] and an increased INR [possibly study drug-related]); one patient experienced increased aspartate aminotransferase that was probably related to the study drug. AEs leading to drug discontinuation occurred in one patient in the dose-expansion cohort (an abnormal liver function test [possibly study drug-related] and bilateral pleural effusion, constrictive pericarditis, and disease progression [all not study drug-related]).

### Laboratory parameters and electrocardiograms

There were no meaningful changes in any clinical laboratory parameters or vital signs over time or any overall shifts from baseline in hematology or laboratory tests.

There was an increase in QTcF values in some patients. Mean (standard deviation) maximal increase of 47.0 (10.7) ms was reported at the 325-mg dose; at the MTD (525 mg), the mean (standard deviation) maximal increase was 25.8 (16.8) ms in the dose-expansion cohort.

### ASP3026 pharmacokinetics

**Fig. 1:**
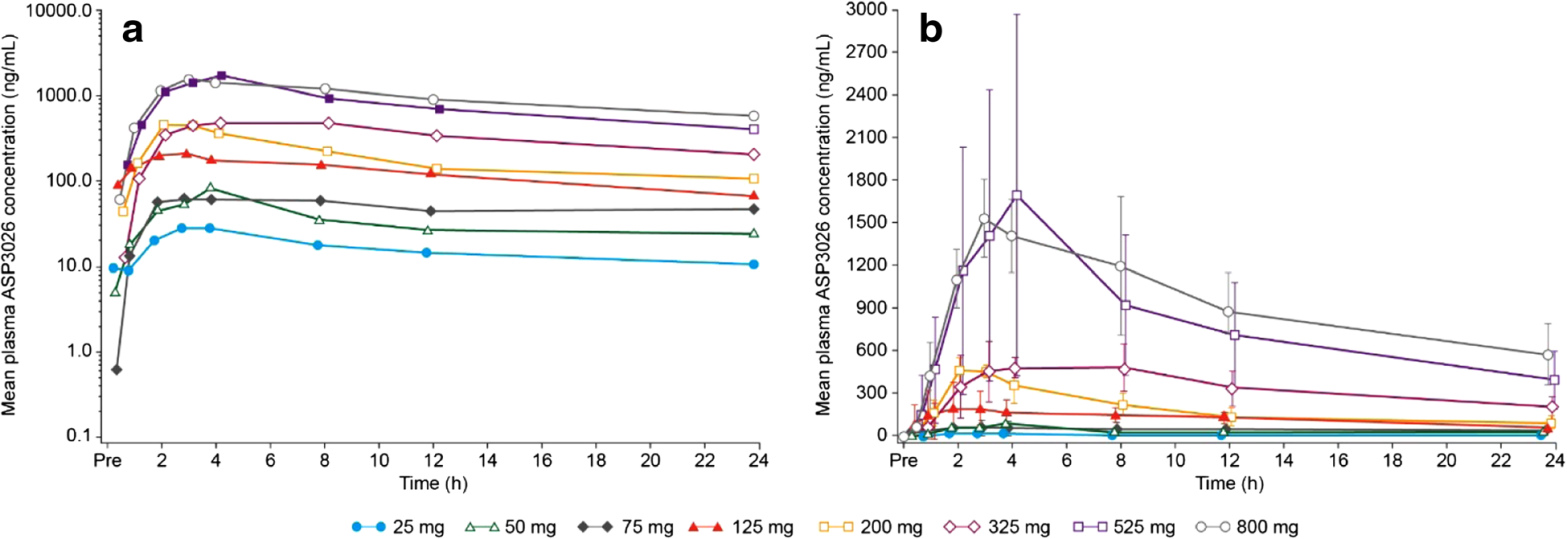
Mean plasma concentration of ASP3026, cycle 1, day 1. **a** Semi-log plot. **b** Linear plot. For patient numbers at each dose, refer to Tables 3 and 4

Mean ASP3026 plasma concentrations by dose are shown in Fig. 1, and PK parameters are provided in Tables 3 and 4. ASP3026 had rapid oral absorption, with a *t*_max_ of approximately 3 h. The mean (standard deviation) accumulation half-life was 25 (37) h (median half-life, 35 h (range, 22–85 h). Steady-state plasma concentrations were reached by day 8 for both the dose-escalation and dose-expansion cohorts. After multiple dosing (day 28), ASP3026 showed both linear and dose-proportional exposure (*C*_max_ and AUC_last_) over the 25- to 800-mg dose range; slope estimates (95 % CI of slope) comparing dose-normalized exposure to dose were 0.170 (−0.018, 0.358) and 0.090 (−0.097, 0.277) for *C*_max_ and AUC_last_, respectively.

**Table 3:** Pharmacokinetic parameters for ASP3026 (cycle 1, day 1)

Dose (mg; once daily)	Number	*C*_max_ (ng/mL)	*t*_max_ (h)^a^	AUC_24_ (ng h/mL)
Dose-escalation cohort				
25	4	32.0 (10.2)	3.0 (0.5–4.0)	378 (104)
50	3	99.7 (56.6)	3.0 (2.0–4.0)	846 (225)
75	3	87.6 (40.3)	8.2 (2.0–24.2)	1155 (442)
125	4	261.7 (131.1)	5.0 (1.0–8.0)	3000 (860)
200	4	490.5 (68.6)	2.5 (2.0–3.0)	4585 (1310)
325	3	586.2 (109.7)	3.0 (3.0–8.0)	7950 (1957)
525	6	1750 (1279)	4.0 (3.0–4.2)	18,543 (10,812)
800	3	1633 (252.7)	3.0 (2.0–8.0)	21,796 (5990)
Dose-expansion cohort				
525	16	961.0 (563.8)	3.1 (2.0–8.0)	11,746 (9063)

Calculated accumulation ratio (AUC_d28, tau_/AUC_24h_ of cycle 1, day 1)

*AUC* area under the concentration–time curve, *C*_*max*_ maximum concentration observed, *NA* not applicable, *t*_*max*_ time of maximum concentration observed

^a^Median (range); mean (standard deviation) for other parameters

**Table 4:** Pharmacokinetic parameters for ASP3026 (cycle 1, day 28)

Dose (mg; once daily)	Number	*C*_max_ (ng/mL)	*t*_max_ (h)^a^	AUC_24_ (ng h/mL)	*t*_½_ (h)
Dose-escalation cohort					
25	3	68.4 (42.3)	3.0 (1.0–8.1)	1038 (335)	36.6 (15.7)
50	3	143 (62.4)	3.0 (2.0–4.0)	2111 (541)	35.2 (18.7)
75	3	352.5 (147.5)	3.0 (2.0–4.0)	5627 (1791)	84.7 (53.9)
125	1	667.9 (NA)	2.1 (NA)	8967 (NA)	36.7 (NA)
200	3	681.8 (104.8)	3.0 (2.1–3.0)	7620 (1699)	21.9 (1.4)
325	3	1159 (856.9)	4.0 (3.0–4.0)	18,764 (17,647)	26.3 (29.1)
525	6	2819 (1681)	3.5 (2.0–4.1)	40,114 (24,479)	27.3 (5.0)
800	1	4854 (NA)	4.0 (NA)	78,081 (NA)	37.9 (NA)
Dose-expansion cohort					
525	15	1331 (813.7)	4.0 (0–4.1)	19,993 (10,552)	24.9 (12.7)

Calculated accumulation ratio (AUC_d28, tau_/AUC_24h_ of cycle 1, day 1)

*AUC* area under the concentration–time curve, *C*_*max*_ maximum concentration observed, *NA* not applicable, *t*_*max*_ time of maximum concentration observed

^a^Median (range); mean (standard deviation) for other parameters

### ASP3026 antitumor effects

In the dose-expansion cohort (crizotinib-resistant *ALK*-positive; 15 NSCLC; one neuroblastoma), the best overall response was PR in eight patients (50 %) and SD in seven patients (44 %) (Table 5). The objective response rate (CR + PR) was 50 % (95 % CI, 25–75 %). Of the eight patients experiencing a PR, six had lung target tumors. The other two patients had the following target tumors: patient 1 had liver and adrenal tumors; patient 2 had pancreas tail, soft tissue peritoneum, paraaortic, and uterine tumors.

**Table 5:** Best overall response to ASP3026 in the dose-expansion cohort

Parameter	Expansion cohort ASP3026 525 mg (*n* = 16)
Best overall response^a^, *n* (%)	
Complete response	0
Partial response	8 (50)
Stable disease	7 (44)
Progressive disease	0
Unable to evaluate	1 (6)
Objective response (complete response + partial response)	
*n* (%)	8 (50)
95 % CI^b^	25–75 %

*CI* confidence interval

^a^Based on RECIST guidelines (v1.1) and International Working Group revised response criteria

^b^Exact CI obtained using Clopper–Pearson method

**Fig. 2:**
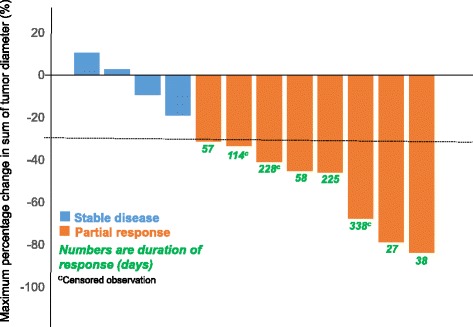
Best tumor response for target lesions in patients treated with ASP3026 in the dose-expansion cohort. Each *bar* represents one patient. The *dotted line* at 30 % indicates partial response. Maximum tumor response for the sum of target lesions is shown. The graph shows 12 patients with evaluable primary tumor diameter changes (one patient was non-evaluable, and for three stable disease patients, the overall response was not based on target lesion)

For patients in the dose-expansion cohort with evaluable tumor diameter, the best tumor reduction is shown in Fig. 2, along with the duration of response that ranged from 27 to 338 days. For those patients with brain metastasis detected at study entry, brain MRI was performed at the end of cycle 2 and then every 2 cycles thereafter per protocol. Progressive or new brain metastasis was considered as PD in addition to RECIST evaluation for extracranial disease. The median progression-free survival in *ALK*-positive patients was 6 (95 % CI, 4–9) months.

## Discussion

Gain-of-function *ALK* gene alterations have been detected in several types of solid tumors, B cell lymphomas and pediatric tumors [2, 3, 22], for which crizotinib has either established or promising clinical efficacy [17]. Several second- and third-generation ALK inhibitors have also shown either established or promising clinical activity for *ALK*-positive NSCLC patients who progressed on crizotinib, supporting the development of potent ALK inhibitors as an effective strategy to overcome resistance to crizotinib [17].

We conducted this first-in-human trial to determine the safety, pharmacokinetic, and antitumor effects of a novel second-generation ALK inhibitor ASP3026. Overall, ASP3026 was well tolerated with no treatment-related deaths. The AE profile in the dose-expansion cohort was similar to that reported for crizotinib [11], with gastrointestinal complaints (nausea and vomiting) being some of the most frequently reported. The constellation of AEs in this small sample set was also similar to other agents in this class, such as ceritinib and alectinib, with gastrointestinal AEs commonly reported [16, 23, 24].

ASP3026 had linear PK parameters and demonstrated dose proportionality over the dose range of 25–800 mg once daily. ASP3026 had good oral absorption, with a *t*_max_ of approximately 3 h; median half-life was 35 h (range, 22–85 h), confirming that once-daily dosing was suitable.

Of the 16 crizotinib-resistant *ALK+* subjects (15 NSCLC and one neuroblastoma) who received 525 mg ASP3026, eight (50 %) achieved PR and seven (44 %) achieved SD at 8 weeks. When restricted to *ALK-*positive NSCLC patients, the PR and SD rates were 8/15 (53 %) and 6/15 (40 %), respectively. Although caution is warranted due to a lack of head-to-head comparison, this tumor response rate is comparable with those observed in other second-generation ALK inhibitors, such as ceritinib and alectinib, in crizotinib-resistant NSCLC patients [16, 24].

Our study has several other limitations. First, although our study was designed to allow the enrollment of patients with *ALK*-driven advanced tumors other than NSCLC, only one patient with advanced neuroblastoma, who had a commonly detected *ALK* F1174L mutation [19–21] and who progressed on prior crizotinib, was identified during the enrollment period. This is because routine molecular testing for other types of tumors did not, and has not, become standard clinical practice. Secondly, multiplexed genomic testing, such as a targeted resistance mutation panel of the ALK kinase domain and targeted next-generation sequencing, was not required at study entry for determining molecular mechanisms of resistance at disease progression to crizotinib. This is unlikely to affect our result. Although different resistance mutations may confer variable responses to subsequent ALK inhibitor therapy [15, 17, 25], most second-generation ALK inhibitors, such as ceritinib and alectinib, as well as ASP3026, have strong efficacy against both secondary mutations in the ALK tyrosine kinase domain and wild-type *ALK* gene amplification [16, 24]. Nevertheless, with the increasing use of clinical molecular profiling tests at treatment resistance in patients with advanced malignancies, individualized treatment beyond a second-generation ALK inhibitor should be based on the assessment of molecular mechanism of resistance.

## Conclusions

The second-generation ALK inhibitor, ASP3026, showed clinical activity in patients with *ALK*-positive solid tumors, especially NSCLC, with half of the patients achieving partial response and a favorable safety profile with a MTD and R2PD of 525 mg daily.

### Ethics approval and consent to participate

The study was conducted in accordance with all applicable regulatory requirements and had institutional review board approval prior to study initiation at participating institutions. Written informed consent was obtained from all patients prior to the initiation of any study-specific procedures.

### Consent for publication

Not applicable.

## Abbreviations

AE: adverse event
ALK: anaplastic lymphoma kinase
AUC: area under the concentration–time curve
*C*_max_: maximum concentration
CR: complete response
ECOG: Eastern Cooperative Oncology Group
MTD: maximum tolerated dose
NSCLC: non-small cell lung cancer
PD: progressive disease
PKs: pharmacokinetics
PR: partial response
RP2D: recommended phase 2 dose
SD: stable disease
TKI: tyrosine kinase inhibitor
*t*_max_: time to maximum concentration
